# Practice to preach self-regulation: Use of metacognitive strategies by medical teachers in their learning practices

**DOI:** 10.12669/pjms.35.6.550

**Published:** 2019

**Authors:** Shabana Ali, Raheela Yasmeen

**Affiliations:** 1 Dr. Shabana Ali, M Phil (Anatomy), MHPE. Associate Professor of Anatomy, Riphah International University, Rawalpindi, Pakistan; 2 Dr. Raheela Yasmeen, MHPE. Professor of Medical Education, Riphah International University, Rawalpindi, Pakistan

**Keywords:** Metacognition, Self-regulation, Learning, Motivation

## Abstract

**Objective:**

To explore use of metacognitive skills by medical teachers in relation to Zimmerman’s model of self-regulation.

**Methods:**

A qualitative phenomenological study was conducted at Riphah International University from February 2017 to August 2017. A semi structured interview of ten medical teachers was planned to uncover the lived experiences of selected teachers to demonstrate how these teachers use metacognitive strategies in different phases of learning. Data was collected by asking five open ended questions after expert validation. Data was analyzed by using N-Vivo software.

**Results:**

Total eight themes were extracted. For prediction and planning three themes, brainstorming, making concept map and sufficient time required were isolated while teachers selected learning objectives and level of students for resource selection while for motivation theme selected was previous experience. Two themes, self- questioning to improve the learning and extra effort required to meet the timeline were isolated for monitoring and reflection during and after learning for evaluation of learning process.

**Conclusion:**

During forethought phase, medical teachers predict their learning process through learning objectives and plan after brainstorming to make a concept map and use suitable learning resources. During learning, they monitor learning process through self-questioning and put extra-effort to meet the deadlines. During and after learning, teachers reflect on their performance.

## INTRODUCTION

Teaching is a deliberate, goal-directed activity that occurs in a constantly changing environment.^1^ The “teaching task” cannot be concluded effectively without learning. A teacher conducts interactive lectures and small group discussions after developing suitable learning support materials such as simple handouts, multimedia programs and simulators to help students. Content preparation is a self-directed, ongoing activity in which teachers explore and assimilate knowledge.^2^ Learning process in medical teachers needs to be regulated to handle the task along with ups and downs of their profession.^3^ They must constantly foster their motivation, commitment, fulfilment and efficiency.^4^ Self-regulation comprises a general time-ordered sequence, an individual passes through while doing a learning task.^5^ Contemporary medical education considers that teachers are like other learners who use many strategies^6^ to achieve the learning goals and teachers can utilize self-regulation just like their students.^2^

**Fig. 1:**
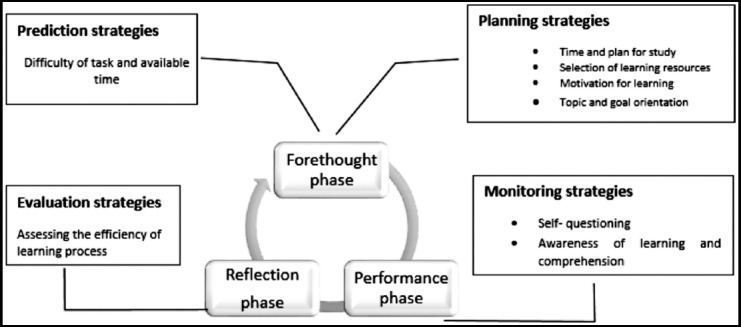
Application of metacognitive strategies in three phases of Zimmerman’s model of self-regulation of learning.

According to Zimmerman’s model of self-regulation, for each teaching session, learning (Fig. 1) occurs in forethought, performance and reflection phase^7^ where teacher plans for learning, execute learning process followed by evaluation of learning process. Successful planning and preparation necessitate proper use of metacognitive strategies

Metacognition is a combination of metacognitive knowledge (cognitive skills), and metacognitive control/ strategies. The “metacognitive knowledge” helps teacher to create meaningful links among thinking processes, the topic to be learnt and prior knowledge. The metacognitive strategies explain one’s ability to use metacognitive knowledge to attain various cognitive objective.^8^ It is the precise evaluation of what is already known and what is yet to be known.^9^ These strategies enhance permanent learning, develop inquisitive skills, nurture social skills and hence, the success in learners. To achieve high degree of metacognition, a teacher must learn to choose, apply and modify an appropriate strategy to improve their learning process for a given learning task.^10^

A teacher can apply four types of metacognitive strategies during learning: prediction, planning, monitoring and evaluation. Prediction skills are used to think about learning objectives, difficulty of task and available time. Planning strategies enable learner to achieve the learning goal through setting their sub goals, choosing appropriate cognitive strategy and activating relevant prior knowledge for future organization and comprehension. Monitoring strategies help to find gaps in their learning process. Evaluative strategies allow the learners to bring learning behavior in line with goal/ standard.^8^,^11^

Although teaching is thought be a secondary activity in relation to the medical profession^12^ but a medical teacher who is well equipped with self -regulation skills will be able to train his trainees through modelling the same behavior.^13^ It will help those borderline students who stop learning before getting mastery of content. In medical education, trainees shall get parallel training for role of metacognition in learning, clinical reasoning and enhancement of performance.^14^ The goal of clinical teaching is “clinical reasoning” 15 and this skill develops with practice provided a feedback loop is used by the practitioner to improve his skills. Therefore, it is imperative to explore the extent to which medical teachers use metacognitive control/ strategies during their learning process. Thereby, we aimed to explore the metacognitive skills displayed by medical teachers. The study was planned as an attempt to assimilate use of metacognitive strategies by medical teachers during their learning process in relation to Zimmerman’s model of self-regulation.

## METHODS

A qualitative phenomenological study was conducted at Riphah International University after approval from ethical review board from February 2017 - August 2017. (Riphah/IIMC/ERC/17/0290) A semi structured interview of ten medical teachers was planned to uncover the lived experiences of selected teachers to demonstrate how these teachers use metacognitive strategies in different phases of learning while facing professional, social and professional pressures. Both male and female preclinical and clinical teaching faculty, assistant professor to professor, with at least 10 years of teaching experience were included. Purposeful sampling technique was used through concept sampling strategy to understand the phenomena of learning in medical teachers having either no qualification or CHPE and MHPE. Concept sampling strategy was selected to uncover metacognitive aspect of self-regulation of learning in medical teachers. All respondents were senior faculty members. Five open ended questions (Table-I) were designed in initial phase. Expert validation was done by sending these questions to three medical educationists. Before data collection we reflected upon our personal beliefs about teaching and learning for teaching sessions to identify personal judgments during data collection and analysis.

**Table I:** Questions for semi structured interview.

1. How do you plan for your lecture?
2. How do you choose your learning resources?
3. What motivates you for learning?
4. How do you monitor your learning process?
5. How do you assess your learning process?

N Vivo software was used for data analysis by using footsteps of Edmond Husserl who described phenomenology as “*study of personal experience and requires a description or interpretation of the meanings of phenomena experienced by participants in an investigation*”.

During textual data analysis, data reduction process was used to form the open codes. The codes were saved in nodes and were categorized under themes only after reading each code in that node. Thematic analysis was carried out to answer the research question. Frequency (descriptive) of each code was noted and constant comparison of statements was done to create a theme at “manifest level” after multiple readings content. Afterwards, these themes were gathered under respective phases from Zimmerman’s framework of self-regulation of learning. Structural analysis was done while explaining the themes to scaffold the phenomena of learning to reach common essence. After data analysis, eight themes were extracted (Table-II).

**Table II:** Themes extracted after data analysis in relation to Zimmerman’s Model of self-regulation.

Phase of Learning	Metacognitive strategies	Themes	Codes
Forethought	Prediction and Planning	Brainstorming	Plan, Outline, Brainstorm, concept map, sequence, arrangement, Time
		Making concept map	
		Sufficient time required	
	Learning resource	Learning resources according to learning objectives and level of students	Learning objectives, Level of student, Need of student, Intrinsic interest
	Self-motivation beliefs	Previous experience plays a prominent role in learning	Experience, Intrinsic interest, Subject domain
Performance phase	Monitoring	Self- questioning to improve the learning	Summarize, recap, question, learning, check
		Extra effort required to meet the timeline	Commitments, social, professional,
Evaluation	Reflection	Reflection during and after learning	Notice, feel, think assess, Reflection, monitoring

## RESULTS

For planning, three themes were isolated. The theme “brainstorming” explained respondent’s views about planning as an elementary factor for effective teaching. Respondent 8 said: “I do my brainstorming about what students can ask from me and to make the lecture more interactive”. Teachers believed in making concept maps/outline of teaching session to foresee all possible class events. Teacher claimed “First I will make a concept map, what I will teach them in a sequence then I will make reading from different sources. Theme “Sufficient time required” explains teacher’s demand for adequate time for planning. Same respondent demanded that “Time table should be given to about 7 to 8 days before the exact commencement of the lecture.

Learning resource as per level of students and learning objectives help teachers to choose the reading material. Choice of resources vary according to topic, content and subject matter. Respondent 4 replied: I use different resources. I like to use text books because they are more authentic. I also use internet material if I found something missing or if I need more elaboration. While Respondent 7 revealed: “The most important goal is student’s level, I want them to understand. I use internet, my course books umm new addition, new articles. This all depends upon learning objectives.”

Teachers reflected on their “previous good experience as a source of motivation” for active planning in next lectures while a bad experience in one class demotivated for next teaching session. Respondent 6 replied: I think it comes from your previous experience of teaching same class. Respondent 8 responded: whenever I take PBL session, if something goes wrong, I remember that experience. I try to plan to overcome that difficulty which happened previously done.

Self- questioning to improve the learning. Respondent 7 said “to make my lecture more interactive, I recap the new concept and think what questions my students can ask from me?” “Extra effort required to meet the timeline” explains their practice to monitor their learning process. Respondent 2 added: “Yes, I do try to monitor, sometimes I cannot make presentation. I try to make sure that I catch up the next day but usually I skip my other commitments to prepare the lecture first”.

The theme “Reflection during and after learning “described both monitoring and evaluation of learning process. According to respondent number 1: “I do reflect, and I do make sure when I do next time its lot better than the earlier version.” Respondent number 7 said: I analyze my learning effort and compare them with student’s attitude in class and try to identify possible measure which may improve student participation in class”.

## DISCUSSION

Effective teachers are self-regulated learners who can stimulate their beliefs to take suitable actions leading to successful execution of their professional learning tasks. These actions need regular practice of metacognitive strategies. A self-regulated teacher displays good cognitive functioning, teaching with metacognitive elements and construction of knowledge before and after teaching session.^2^ This exercise improves their teaching practices as well as inculcates critical reasoning skills in our future doctors. Therefore, modelling of this behavior is mandatory for brushing up the learning skills of learners.

Lesson planning is a deliberate activity^8^ which lies at the heart of effective teaching in any setting. Medical teachers use this creative process to tailor their learning to combat busy schedules at hospitals and this workload is increased by agonistic action of social and personal commitments. Therefore, based on their prediction skills, teachers demanded to have sufficient time before the commencement of their session. It also sensitizes them about difficulty of task. Therefore, they brainstorm with their colleagues to analyze the given learning task and make suitable plan to go through their tough schedules accordingly. Brainstorming is a useful tactic of active learning because learners take charge for their own learning.^16^ Self-regulated teachers are proactive agents who can design appropriate instructional practices and control teaching environment and conditions.^2^,^17^ Brainstorming strategies facilitate their comprehension ability^18^ as well as another study proved effectiveness of brainstorming in developing students’ creative thinking skills in learning nutrition.^19^ Therefore, brainstorming informs them about their metacognitive knowledge which facilitates a deliberate effort in designing a clear outline/ concept map of their teaching session. Thinking on and after action along cyclic self-regulation cycle is an important step for comprehensive learning.^20^

Resource selection helps to develop a useful teaching material. Medical teachers are focused to teach what is required and according to learning objective.

In performance phase, learning experience needs monitoring of learning to compare the progress of learning against goals/ standards for future actions.^21^ Medical teachers believe in self questioning which needs a thorough exploration of given concept, imagination of expected class room environment and learning requirements of their students.^22^ It facilitates concept building through activation of prior knowledge and high order comprehension in their students which improves retention of knowledge. In case of any unforeseen situation, metacognitive teachers take remedial measures, skip some other commitment and give priority to their learning task. The self-regulated learner verbalize clear learning intentions and calculate the effort they need for completion of task and know whom they can ask for help.^23^

Medical teachers get their motivation from good previous experience. Motivational beliefs are associated with self-efficacy^24^,^25^ which enable a learner to use elaboration and metacognitive strategies along with time and environment management.^26^ Knowledge and beliefs about motivation effect the selection of goals, learning strategies and one’s determination in a specified task.^20^,^25^ When learners are motivated to learn, they dedicate the necessary time and energy needed to learn.^27^

During evaluation phase, reflection is observing own actions, achievements and failure and is a useful tool for improvement in learning practices.^28^ Self- evaluation occurs through comparison of present performance against some personal standards^29^ leading to effective teaching and medical teachers prefer to evaluate their learning both during preparation and after their teaching session. This practice helps them to identify their strengths and weaknesses that must change to bring academic achievement in their students in future.^29^

The study was carried out in a private institute involving both preclinical and clinical teachers. In future more studies on same topic with gender discrimination, exploring preclinical and clinical teachers separately can be planned.

## CONCLUSION

During forethought phase, medical teachers use brainstorming to make a concept map of their task during planning. They predict difficulty of their task through learning objectives and level of student. Their previous experience motivates them to learn and during learning performance, they use self-questioning to enhance their higher order thinking. During evaluation phase, they reflect upon learning to look back at their concept and to identify areas for more effort.

### Authors’ Contribution:

**SA:** Drafting the article or revising it critically for important intellectual content .Is also responsible for integrity of the research

**RY:** Final approval of the version to be published.
